# Devolution and its effects on health workforce and commodities management – early implementation experiences in Kilifi County, Kenya

**DOI:** 10.1186/s12939-017-0663-2

**Published:** 2017-09-15

**Authors:** Benjamin Tsofa, Catherine Goodman, Lucy Gilson, Sassy Molyneux

**Affiliations:** 1 KEMRI Wellcome Trust Research Programme, KEMRI Centre for Geographic Medicine Research Coast, P.O. Box 230-80108, Kilifi, Kenya; 2 Global Health Department, Faculty of Public Health and Policy London School of Hygiene and Tropical Medicine, London, UK; 3 Health Economics Unit, School of Public Health and Family Medicine, University of Cape Town, Cape Town, South Africa; 4 Centre for Tropical Medicine and Global Health, Nuffield department of Medicine, University of Oxford, Oxford, UK

**Keywords:** Decentralisation, Devolution, Governance, Health workforce management, Commodities management

## Abstract

**Background:**

Decentralisation is argued to promote community participation, accountability, technical efficiency, and equity in the management of resources, and has been a recurring theme in health system reforms for several decades. In 2010, Kenya passed a new constitution that introduced 47 semi-autonomous county governments, with substantial transfer of responsibility for health service delivery from the central government to these counties. Focusing on two key elements of the health system, Human Resources for Health (HRH) and Essential Medicines and Medical Supplies (EMMS) management, we analysed the early implementation experiences of this major governance reform at county level.

**Methods:**

We employed a qualitative case study design, focusing on Kilifi County, and adapted the decision space framework developed by Bossert et al., to guide our inquiry and analysis. Data were collected through document reviews, key informant interviews, and participant and non-participant observations between December 2012 and December 2014.

**Results:**

As with other county level functions, HRH and EMMS management functions were rapidly transferred to counties before appropriate county-level structures and adequate capacity to undertake these functions were in place. For HRH, this led to major disruptions in staff salary payments, political interference with HRH management functions and confusion over HRH management roles. There was also lack of clarity over specific roles and responsibilities at county and national government, and of key players at each level. Subsequently health worker strikes and mass resignations were witnessed. With EMMS, significant delays in procurement led to long stock-outs of essential drugs in health facilities. However, when the county finally managed to procure drugs, health facilities reported a better order fill-rate compared to the period prior to devolution.

**Conclusion:**

The devolved government system in Kenya has significantly increased county level decision-space for HRH and EMMS management functions. However, harnessing the full potential benefits of this increased autonomy requires targeted interventions to clarify the roles and responsibilities of different actors at all levels of the new system, and to build capacity of the counties to undertake certain specific HRH and EMMS management tasks. Capacity considerations should always be central when designing health sector decentralisation policies.

## Background

Decentralisation is argued to promote community participation and accountability, and enhance technical efficiency and equity in the management of public resources. Within the health sector, decentralisation has been a recurring theme in health system reforms for several decades [1, 2]. The implementation of decentralisation polices within the health sector has adopted a wide range of modes and forms, determined by the nature and structure of the sub-national level entity to which responsibility is transferred. However, irrespective of the form, the final effects of decentralisation reforms have been influenced by many internal and external factors including the reasons or drivers for decentralisation, and the country’s political context [1, 3–6]. In practice decentralization involves shifting power and authority over the management of public resources from national to sub-national levels of government. This makes it a highly political reform, though its political nature and context are rarely analyzed in empirical studies [1, 4, 7, 8].

Human Resources for Health (HRH) and Essential Medicines and Medical Supplies (EMMS) are two critical building blocks of health systems. Considering that the two attract a substantial amount of total health system funding, they often generate contention during the design and implementation of health sector decentralization policies [2, 9]. However, even with the acknowledgement of the central role of HRH and EMMS, decentralization policy formation and debate mainly focuses on financial resource allocation, financial management and reporting, with HRH and EMMS management plans rarely featuring [2, 10].

In June 2015, we carried out a systematic search of published empirical studies on the effects of decentralization on HRH and EMMS management in LMICs, published in English language between 1983 and December 2014 which identified 14 articles on HRH, and 7 on EMMS. The studies described a wide range of both positive and negative effects on HRH and EMMS management in LMICs. On HRH management, several studies reported decentralization being associated with better attraction and retention of lower cadre staff, but poor attraction of specialized health workers [11–15]. In Tanzania for example, after undertaking decentralization for all HRH management functions to the district level, rural districts were unable to attract and retain highly skilled staff such as medical specialists, leading the country to re-centralize some of the HRH management functions [12, 16]. Some studies suggested that certain HRH management functions, including recruitment and distribution of highly specialized health workers, in-service training, and management of staff salaries, are best managed centrally [11–13, 17]; while other functions like staff appraisals, promotions, recruitment and deployment of lower cadre health workers are best handled in decentralized units [12, 14]. Another commonly reported HRH management problem linked with decentralization has been frequent delays and disruptions in payments of staff salaries; and challenges in managing in-service training and other career progression initiatives [12, 14, 18]. In addition, several studies identified challenges in the management of the responsibility transfer process from central level to decentralized units, in the early stages of decentralization. This has often been associated with confusion, fear and anxiety on the part of health workers. In many instances, these HRH management challenges have resulted in low staff morale, industrial action like strikes and mass resignations [12, 13].

On EMMS management, the literature shows that many countries with decentralized health systems retained most EMMS management functions under central control. In most cases it was argued that the central level had better capacity to undertake quantification of EMMS, obtain economies of scale associated with bulk purchases, and monitor and reinforce quality of drugs and commodities supplied [10, 11, 17]. However, where EMMS management was decentralized, there was been some documentation of better budgetary allocation for commodities, leading to better servicing of commodity [19] orders at facility level, for example in Ghana and Guantemala [10].

As part of the implementation of the 2010 constitution, the Government of Kenya in 2013 adopted a devolved government system with 47 semi-autonomous county governments, with significant decision making autonomy, and minimal central level control [20–22]. The design and implementation process of the devolved government system was largely driven by a political push to address real and perceived long-term political challenges of marginalization and inequitable resource allocation in the country [19, 23]. Within the health sector, the constitution outlined that all health service delivery functions, including the procurement of EMMS and management of HRH, would be assigned to county governments, while the national Ministry of Health (MoH) was assigned the roles of health policy and standards formulation, pre-service training for health workers, and management of national referral services. A detailed breakdown of the functions assigned to national and county governments is found in the schedule 4 of the constitution [20].

The constitution outlined a five-year plan for establishing county government structures and progressive transfer of functions. This was to begin with the establishment and capacity building of county level structures between 2010 and 2013, and progressive transfer of functions over a 3 year period from 2013 [20], facilitated by a Transition Authority [24] (Tsofa B, et al.: How does decentralisation affect health sector planning and financial management? A case study of early effects of devolution in Kilifi County, Kenya, submited). However, once they were elected into office in early 2013, the county governors began to agitate for an immediate transfer of all county level functions. The president in June 2013 succumbed to the pressure from the governors and directed that all county functions be devolved immediately, though at that time most counties had not established structures to undertake these functions (Tsofa B, et al.: How does decentralisation affect health sector planning and financial management? A case study of early effects of devolution in Kilifi County, Kenya, submited) [25].

Kenya thus provides an ongoing opportunity to examine devolution of these key health sector management functions of HRH and EMMS. In addition to contributing to the literature on decentralization effects on HRH and EMMS management, this paper uniquely analyses the broader political context within which the devolution reform was implemented, and analyses health sector devolution effects as they played out during the process of implementation.

## Methods

This paper presents data from a broader health systems governance study that has been analyzing the effects of implementation of devolution of the health system in Kenya [21, 25, 26]. The study established a health system governance “learning site” in Kilifi County to examine various questions within the context of devolution in Kenya. A ‘learning site’ is an embedded approach to health policy and systems research, where researchers and health managers in a given setting over a long-term relationship of continuous interactions and reflections develop specific health system governance questions, and work towards answering them together. HRH and EMMS were selected as tracer topics within this study [25, 27].

Kilifi county has a population of approximately 1.2 million people and covers an area of 12,246 km^2^. About 74% of the population live on less than one dollar a day. It is one of the counties in the Coastal region of Kenya believed to have been the historical champions of devolution in the country [28]. Both BT and SM are part of the Kilifi learning site, while LG is part of sister learning site in Cape Town, South Africa [29].

### Conceptual framework

**Fig. 1:**
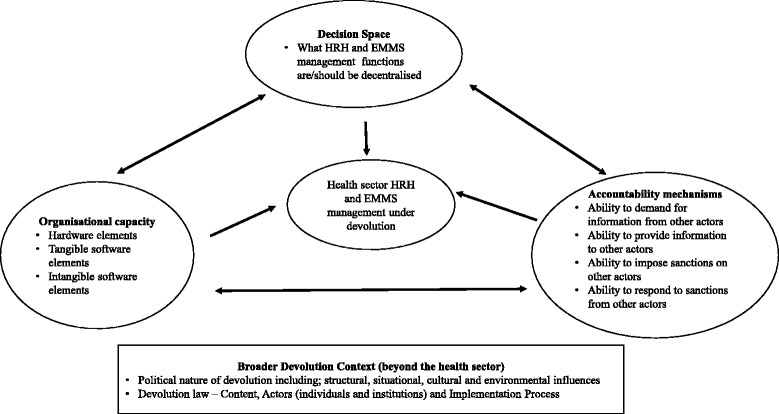
Study conceptual framework

Drawing on principles from the principal-agent theory [30], Bossert (1998) developed a *decision space framework* for analysing health sector decentralisation. In his decision space framework, Bossert described the ‘principal’ as a central government entity with a health service delivery mandate that it transfers to an ‘agent’, which he describes as a peripheral entity. Bossert argued that in decentralization policies, there is always a range of effective choices that the ‘agent’ is allowed by the ‘principal’ to make. He furthers argued that these choices though often defined within laws and guidelines; however, the ‘actual’ choices that the ‘agent’ ends up taking often include ‘bending the law’ because of the inability of the ‘principal’ to reinforce adherence of the rules. It is this total range of choices that the ‘agent’ ends up taking as outlined in the rules, and due to their ability to ‘bend the rules’ that Bossert described as *decision space.* This framework has been applied in several studies of health systems decentralization in low and middle income countries (LMICs) [5, 18, 31, 32]. More recently, Bossert and Mitchell (2011) have further argued that in any decentralized units, *decision space* often interacts with and is affected by *organizational structure and capacity* of these units, and the *accountability* structures and mechanisms of the designed decentralization system [33]. Informed by the literature and our own study findings, we further adapted this framework (see Fig. 1) by incorporating the *broader political context* as an essential element influencing the interactions between decision space, organizational structure and capacity, and accountability mechanisms (Tsofa B, et al.: How does decentralisation affect health sector planning and financial management? A case study of early effects of devolution in Kilifi County, Kenya, submited). We utilized this framework to develop and structure our data collections tools, and in this paper, we draw on this framework in discussing our findings.

### Data collection and analysis

As part of the learning site activities [34], we conducted participant and non-participant observations in Kilifi County between December 2012 and December 2014. We also held regular quarterly reflective practice sessions among the learning site researchers, and with local health managers lasting between 3 and 3 hours each session [21]. We maintained field notes in form of a diary for the observations, and the reflective practice session discussions were audio taped and transcribed. In addition, during the same period, BT conducted non-participant observations at the national level MoH, while at the same time systematically monitoring and tracking the broader national level implementation of devolution and ongoing implementation debates, through print and electronic media, and documenting key events in a diary (Tsofa B, et al.: How does decentralisation affect health sector planning and financial management? A case study of early effects of devolution in Kilifi County, Kenya, submited). We further identified and reviewed all key documents relating to the implementation of the devolved government structure in Kenya generally, and planned health sector implementation specifically. These observations and the document review were supplemented by 28 semi-structured interviews, each lasting about 1 h, in English with a wide range of purposively selected participants (Table 1). The interview guide covered questions on all the elements of our conceptual framework (Fig. 1). The participants represented a wide range of actors who had a key role in the implementation of the devolved government, and within the health sector specifically at the national, county and sub-county level, at the Kilifi learning site.

**Table 1:** Summary of type and numbers of documents reviewed and participants interviewed at different levels

Documents reviewed	
Broader decentralization laws/policy documents	The 2010 Constitution - 1 Transition to Devolved Government Act 2012–1 County Government Act 2012–1
Health sector specific implementation policies	Ministry of Health Functional Analysis and Transfer (FACT) policy - 1 The Kenya Health Policy 2013–2030 - 1 The Kenya Health Sector Strategic Plan 2013–18 - 1
Range of participants interviewed	
At national level	National Ministry of Health (MoH) - 3 Non-government health development partners - 2 UN agencies representatives - 2 Commission for the Implementation of the Constitution The Transition Authority – 1 National Assembly Committee on Health - 1
In Kilifi county	County Department of Health (CDoH) Managers - 5 County Public Services Board - 1 County Assembly (CA) - 1 County Transition Authority coordination office – 1 Sub-County Health Managers- 2

BT’s previous experience in the health sector, and his engagement with the MoH at county and national level during the data collection phase provided him with a unique insider perspective, with access to information and operations of the health system both at national and Kilifi County level that would not be accessible to purely external researcher [35]. To strengthen objectivity in the interpretation of his observations, regular formal reflective sessions were carried out with the other research team members to allow for group reflections on the findings [25].

The document review (see Table 1) provided a broader background understanding of the goals and expectations of the devolved government system within the Kenyan health sector, as well as specific information on HRH and EMMS, with information summarized using a content extraction template. The interviews drew on the formal and informal observations and discussions over a long period, and involved discussion of both general experiences of health sector implementation of decentralization, as well as specific questions focused on health workforce and health commodities management.

We imported all the data into NVivo 9 software and used the thematic framework approach for data analysis [34]. The initial themes were guided by the elements of our conceptual framework, which we refined as we familiarized ourselves with the data.

## Results

We present our results, first for HRH, and secondly for EMMS. For each tracer, we begin by providing an overview of the organization and management of these functions prior to devolution, and a summary on how they were supposed to be organized under devolution in theory (policy on paper), before turning to key issues arising in the actual implementation process and experiences of actors (policy in practice).

### HRH management

#### Intended structures and implementation process for HRH management under devolution

Before devolution, the national Public Services Commission (PSC) served as the overall employer of all government workers in the country, including health workers. Its role was to provide overall guidelines and oversight for strategic human resource development and management in the public sector, while the routine operational human resource management functions including recruitment, appraisal, promotions, discipline, in-service training and payment of salaries were delegated to the respective government ministries, including the national MoH for all health workers.

Under the devolved government system, the PSC is mandated to provide employment for national government employees, and oversight of the entire public service both at national and county level. At the county level, the constitution provided for the establishment of County Public Service Boards (CPSBs) in each county that would serve as the overall employer of all public servants in that county.

Public servants performing devolved functions at the time of the general elections on March 2013 were to be seconded to the county governments where they are working, to be formerly deployed or transferred to those county governments once the county human resource management structures were established. Within the health sector, national MoH in liaison with the constitutionally established Transition Authority was to undertake a human resource capacity assessment for the counties (Tsofa B, et al.: How does decentralisation affect health sector planning and financial management? A case study of early effects of devolution in Kilifi County, Kenya, submited). The Transition Authority was to further work to build capacity for all CPSBs to enable them to undertake the assigned pubic service management function, and then work with respective ministries to transfer all staff working in the counties from the national government to the respective CPSBs.

#### Implementation experiences and outcomes of HRH management under devolution

##### Lack of clarity over HRH management roles at county level

Beyond overall responsibility for the management of county government employees, it was unclear what specific operational human resource management responsibilities the CPSB would have for the respective technical county departments, leading to a lack of clarity over HRH management responsibility between the CPSB and the County Department of Health (CDoH). There was also lack of clarity over which structures and institutions at national and county level would be responsible for specific welfare aspects of health workers, including in-service training and career progression; and how inter-county transfers for health workers would be managed. Some of these confusions were expressed during the interviews:

…Things were not also very clear with this human resource management issue; it’s not clear who is to undertake what role…. KII SC 005

…Now the movement is still not very clear to us with, I mean now outside the county. So today we had a meeting with the director but we didn’t discuss it, we just talked about the persons who want to be moved within the county but the question of movement outside the county remained un-answered…. KII SC 002

##### Rushed transfer, dialogue and interim arrangements for HRH management

Given the rushed transfer of devolved functions, and the challenges this brought for HRH management in counties at the time, several health sector stakeholders at national level came together to dialogue on these HRH management challenges. To avert a crisis of counties failing to pay salaries for health workers countrywide due to lack of capacity to undertake payroll management, the national health sector Intergovernmental Relations Forum that brings together the national MoH with the 47 CDoHs, was convened to develop an interim solution. It was agreed that for an interim period of 6 months the national MoH would continue processing and paying salaries for all health workers on behalf of the counties, then invoice the county governments for reimbursements. It was expected that counties would take the 6 months’ interim period to set up their systems, leading to them taking up the role of health worker salary payments.

….[…there were arguments that the (national) government, was anti-devolution and to be seen not to be anti-devolution we said yes, these services must be transferred (immediately) and that is it. The functions were transferred….] KII N 002…..

…..through you know negotiations with county governments, I think they accepted that national government would continue paying salaries of county workers in the interim up to December 2013 as they prepare themselves…] KII N 001……

##### Disruptions, delays, and discrepancies in health workers’ salaries

County governments went ahead to prepare their payroll management systems in readiness for undertaking the role of paying salaries for health care workers by January 2014. However, when they eventually took up this role the initial months were characterized by several challenges including general delays in salary payments, payroll discrepancies and missing allowances; and some staff missing from the payroll altogether.

##### Public participation and accountability in recruitment of senior public servants

The constitution provided an opportunity for public participation in the appointment of senior public servants including Cabinet Secretaries and Principal Secretaries of ministries at national level, and County Executive Committee (CEC) members and Chief Officers of departments at county level. The president (at the national level) and governors (at county level) nominated individuals for appointment into these positions. The nominated individuals’ names were then submitted to the National and County Assembly committees respectively, for a public vetting exercise. Members of the public were invited to present their views in the form of memoranda to the vetting committee, in support of or in contest of the appointment of the nominees. The nominees where then appointed after clearance by the vetting committees. However, at both national and county level, this was seen by many as a public relations exercise as many people felt that members of both the National and County Assemblies did not have the required skills and capacity to undertake meaningful assessment and vetting for these public officers. Secondly there were strong perceptions that both the National and County Assemblies were regularly compromised by their respective executive arms of government, through allowances and other inducements to ‘rubber stamp’ executive decisions and choices.

…. public participation is very weak, very weak; because when you bring a bill, you bring them (Members of the County Assembly) here to take them through … you invite the committee to take them through the Bill. Pay them a sitting allowance; When they get it there at the assembly, they will not raise a finger on it…. KII C 002

##### Political interference and discrimination in HRH management

Political interference and discrimination in HRH management began to be reported in some counties immediately the devolution process began. One national level interviewee highlighted an example where in mid-2013, the national MoH deployed to counties a group of freshly qualified medical doctors who had completed a one-year statutory government internship training as required by law. It was however reported that some of the counties rejected the doctors sent to them because they had come from different tribes or counties from the ones they were posted to.

…It’s tricky, because that has happened in the health sector few weeks ago, Dr. XXX posted out some doctors who had either finished training or internship and some counties rejected them claiming they didn’t come from those counties…. KII N 007

Political interference with health workers was also reported at facility level. During one reflective practice session with sub-county managers, it was reported that one dispensary within a Kilifi sub-county had been closed because community members led by their Area Member of County Assembly had demanded to have the only nurse at the dispensary transferred because she did not come from the local tribe. Sub-county level interviewees reported that this growing level of political interference and victimization raised significant concerns among health workers.

The fear for political interference and victimization, coupled with uncertainty over inter-county transfers, led to many health workers wanting to be transferred back to their ‘home-counties’.

…. up to August last year, there had been so many transfers. People were requesting to move away to go back to their home counties people were in state of panic not sure about how their host counties would treat them so they wanted to go back to their homes…. KII C 008

##### Fears, anxiety, low morale and industrial actions by health workers

**Fig. 2:**
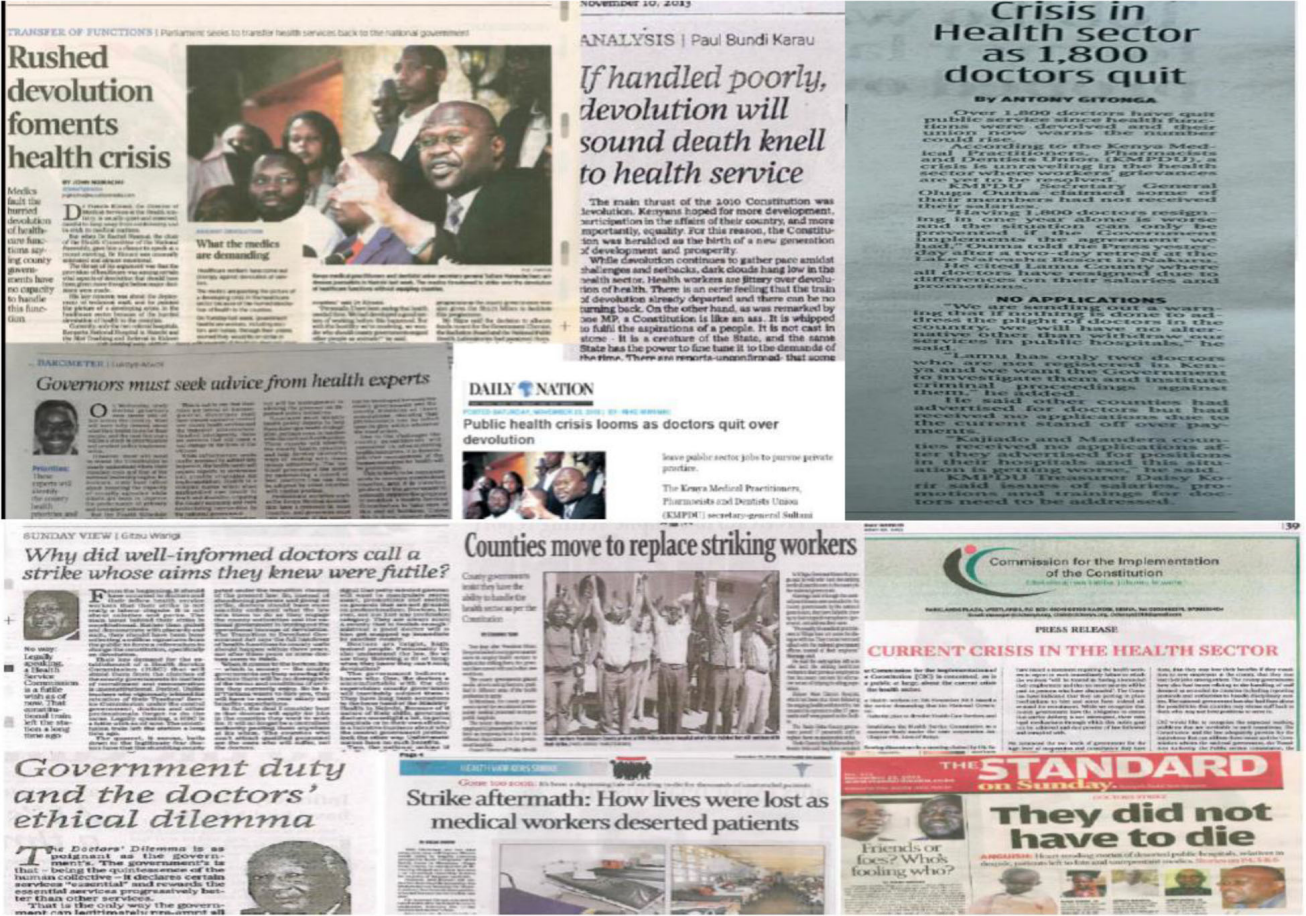
Media coverage of the 2013 health workers strike in Kenya

The multiple challenges and uncertainties over health workforce management highlighted above led to wide-spread fear and anxiety among many health care workers at the time. The concerns included the uncertainty about career progression, uncertainty about inter-county transfers, the increasing political interference over health worker management, and the continued disruptions in salary payments. Owing to these issues, the national media continued to report on cases of mass resignations of health workers countrywide. In late 2013 the three major health worker unions in the country called for a nation-wide strike citing these challenges and pressing for re-centralization of the health service delivery function back to national government. During this strike, which lasted for several weeks, health care workers countrywide resorted to several protest strategies including street demonstrations and social media protests (Fig. 2).

In response to this strike action, the Council of Governors met in Nairobi and took a joint political decision to lay off all striking health workers within their counties. Several counties went ahead to sack all the striking health workers, and re-advertised their positions. This action by county governments, together with the realization by health workers that they could not negotiate for a return to work arrangement centrally, but instead had to do so with respective county governments, softened the position and stand of the health worker unions prompting them to call off the strike in early 2014.

### EMMS management

#### Intended structures and implementation process for EMMS management under devolution

Prior to the roll-out of devolution the Kenya Medical Supplies Agency (KEMSA) - a state corporation under the national MoH - had the statutory mandate for procurement, warehousing and distribution of all EMMS to all government health facilities. In partnership with KEMSA, the national MoH employed a ‘pull’ system for EMMS management and supply for public health facilities where the national MoH would allocate a quota of its annual budget for EMMS to KEMSA. This allocation would then be subdivided between all gazetted government health facilities based on a resource allocation criteria developed by the national MoH. Using the facility allocation, KEMSA would then establish an account for each facility known as ‘drawing rights’ to cover each financial year. Health facilities would then make orders (‘pull’) from their drawing rights at KEMSA on a quarterly basis and KEMSA would service the facility orders while crediting the facility allocation. However, this pre-devolution pull system faced significant challenges, and health facilities often reported prolonged delays in servicing their quarterly orders by KEMSA, and very low refill rates, leading to long periods without essential supplies.

Under devolution, county governments would be the ones to allocate resources for and procure EMMS for government health facilities within their areas. Given the many years of perceived inefficiency in KEMSA, county health managers welcomed these proposals, anticipating that this would give counties greater bargaining power to demand for more efficient supply service from KEMSA. They also felt that if KEMSA was unable to meet their needs, they would be in control and able to decide to out-source through alternative suppliers.

…. I would say devolution is a good thing because we’ve consistently been complaining about KEMSA and now that we have this is actual money. It’s no longer drawing a virtual imaginary figure. This is actual money, then we expect better supply, we expect better supplies for our facilities in terms of quantities and in terms of variety of the medicines… most of the items from KEMSA we will procure from them, then what is not available at KEMSA especially for the hospitals, we’ve asked them to prepare a separate list and for that we will float a quotation…… KII C 004

The process of EMMS management under devolution begins from the health facility level, where the health worker in-charge undertakes needs quantification and submits projected facility requirements to the County Pharmacist. At the county level, the County Pharmacist is responsible for providing technical assistance to facility managers on quantifications. The County Pharmacist then analyses and consolidates the orders from health facilities, prepares purchase orders and submits them to the County Treasury. The procurement department at the County Treasury is charged with the responsibility for the tendering process for the required commodities.

Community involvement and participation in EMMS management is supposed to occur at several levels. First during the priority setting, planning and budgeting process health facility managers are supposed to consult with the health Facility Management Committees (FMCs) made up of community representatives from within the facility catchment area. The facility in-charge is further supposed to consult with the FMCs during the commodity quantification and ordering process. Finally, once the orders have been supplied, the FMCs should be involved in ascertaining that the right orders have been delivered before payments are made to suppliers.

……community involvement especially with the new dispensation, it is very important. We have to sensitize our communities even when the commodities are supplied to the facilities, they should be told that we have received this consignment and in this quantity ……. delivery notes have to be signed by 3 people, the facility in-charge, the facility committee chairperson and another person from the facility management committee … KII C 004

#### Implementation experiences and outcomes of EMMS management under devolution

##### Dialogue, consensus building and interim arrangements

In the early days of implementation of the devolved governments, a health sector Intergovernmental Relations Forum was convened to facilitate dialogue and consensus around EMMS management issues and to develop an interim action plan to address a drug shortage crisis that was at the time being experienced in government health facilities across the country. During this dialogue process, the national MoH with support from health sector development partners, provided funds to procure a 6 months’ commodities starter stock for all government health facilities countrywide. This was done to allow the county government to set up their structures and systems to take up this procurement role.

In addition, following negotiation, an agreement was reached for all counties to make KEMSA the first point of call for their health commodity needs, to gain economies of scale and commodity quality assurance. The counties and KEMSA agreed on a ‘service agreement’ where KEMSA would service the orders from counties within 2 weeks of receiving them, and counties would pay for these commodities upon delivery by KEMSA.

##### Procurement and distribution process of commodities in Kilifi County

The CDoH in Kilifi embarked on the quantification and ordering process for EMMS from the end of 2013 amidst several challenges, including a lack of appropriate technical and infrastructural capacity at facility and county level. Eventually, the first consignment of hospital supplies procured by the county were delivered in February 2014. Though the supplies to lower level facilities were slightly delayed, when they eventually arrived there was a feeling by facility managers that the order servicing by KEMSA has improved compared to pre-devolution.

…They (KEMSA) have now supplied so generally we are not badly off as a hospital. the refill rate was almost to 95%. We did the quantifications that this is what we want and then we raised an LPO and they supplied most of the things we needed……. KII SC 003

In addition, the county government procured drugs for all operational public health facilities within the county, irrespective of their legal registration status. This was different from the old ‘pull system’ where only facilities which had been gazetted were allocated drawing rights for commodities by KEMSA. Considering that the gazettement of health facilities used to be undertaken by the national MoH, and could take several years from the time a facility was operational, health facilities often operated without drawing rights allocations for drugs and other commodities.

**Fig. 3:**
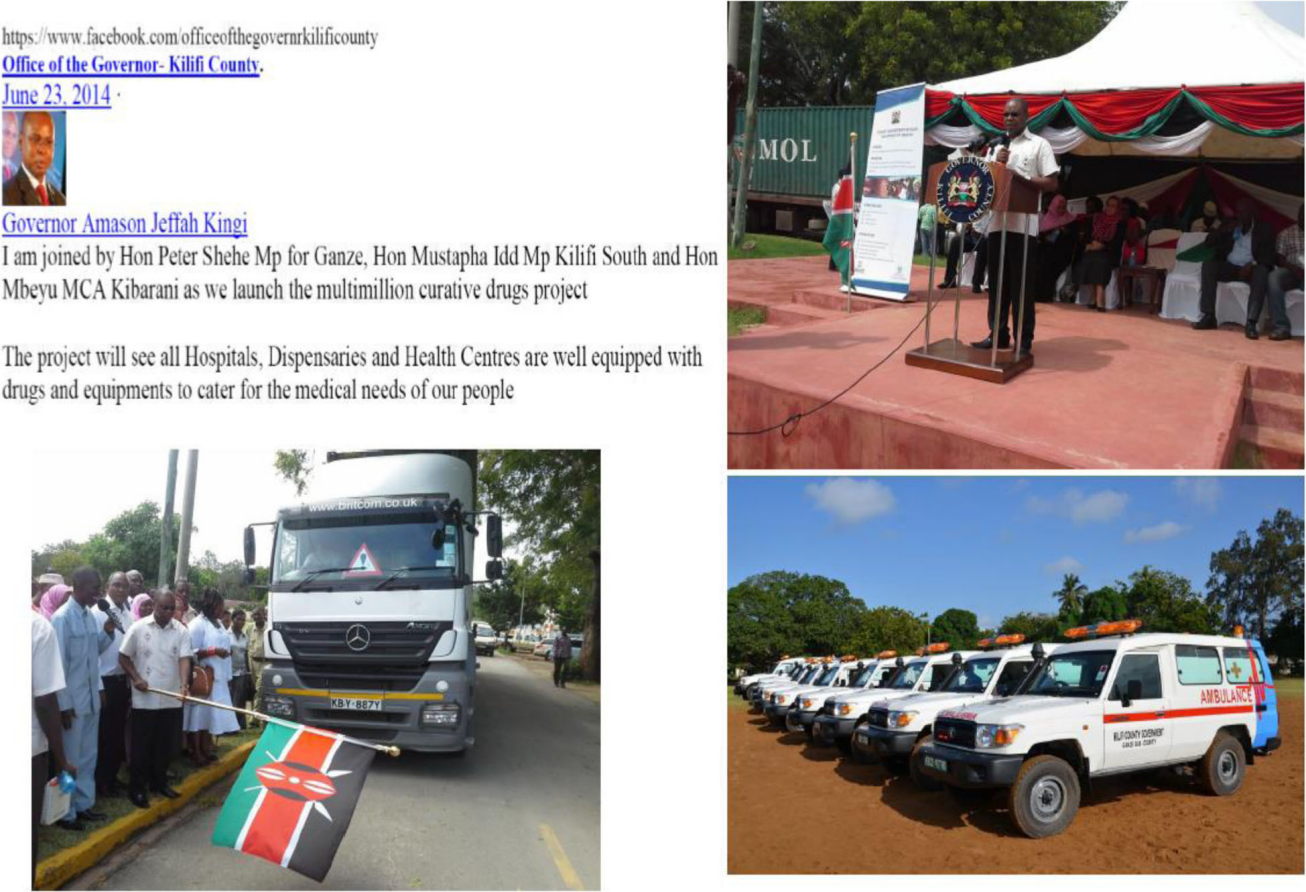
Images of the Kilifi County Governor flagging off drug distribution; and the Kilifi County ambulances

As the counties took up the EMMS management function, there was a visible attempt by several county political leaders countrywide to achieve political visibility over this role. First several counties in their initial days opted to procure ambulances which were perceived to be politically more visible in the eyes of the public and voters, at the expense of procuring essential drugs for their health facilities. This ambulance procurement ‘craze’ by many counties was extensively covered by the national media at the time. Subsequently, when the counties begun to procure drugs, it became increasingly fashionable for governors to organize public rallies every time the KEMSA trucks carrying drugs for health facilities arrived, with a claim of officially ‘flagging off’ the drug supply. This turned what used to be a routine exercise of drug distribution to health facilities into a major political fun-fair in the counties under devolution. Figure 3 is a collection of images illustrating the politicisation of EMMS management in Kilifi County.

## Discussion

Omar [4] argued that the political drivers and context that push a country to adopting decentralized governance arrangements have a major bearing on how decentralization gets implemented in that setting [4]. From our findings, the **political context** in Kenya caused the transfer of devolved health sector functions to be done faster than had been anticipated by most health sector players (Tsofa B, et al.: How does decentralisation affect health sector planning and financial management? A case study of early effects of devolution in Kilifi County, Kenya, submited) [25]. This happened at a time when the county governments had not set up their **organizational structures** and built capacity for these structures to undertake their functions, causing major challenges and disruptions in public sector service delivery. In this paper, we have specifically illustrated how this rapid transfer of functions caused major challenges in the management of HRH, and EMMS at county level.

On HRH management, the rapid transfer of functions before counties had established their HRH management structures meant that they could not undertake key HRH management roles, including payroll management and payment of salaries. However, an interim arrangement was agreed, where national MoH continued to pay staff salaries up to December 2013, and invoiced county governments for reimbursements. When the counties eventually took up this role, payment of staff salaries was often delayed, with numerous pay-roll inconsistencies and discrepancies and some staff totally missing from the payroll. There were also some reported cases of political interference in HRH management across the country. Other challenges included a lack of clarity over key HRH management roles by different players, including management of inter-county transfers, in-service training and career progression for health care workers. These challenges led to observed and reported fear, anxiety, and mass resignations of health care workers across the country; and eventually culminated into a protracted health workers’ strike that crippled the health sector country-wide for several weeks in late 2013.

Similarly, the EMMS management function was affected by the rapid transfer of responsibilities. In the early days after devolution, there were arguments and contestations between national MoH and CDoH country-wide on the role of KEMSA. In the interim phase, national MoH, with funding from donors, supplied 6 months’ worth of buffer stock of drugs for all public health care facilities countrywide as a stop-gap measure to allow for counties to set up their procurement and distribution systems. When the county governments eventually took up this role, there was widespread politicization of the drugs and commodities procurement and distribution within the health sector. Nevertheless, health facilities reported better fill rates whenever drugs were supplied.

Though our findings show that the implementation of the devolved government system in Kenya significantly increased ***the decision space*** for HRH and EMMS management at county level, the ability of counties to claim, and utilize this space was undermined by an initial lack of proper structures and capacity to fully undertake all the HRH and EMMS management functions. However, with time as counties established their structures and built their capacity they did increase their ability to utilize the expanded decision space over these roles. Table 2 below illustrates our analysis of the the shifting county level health sector decision space over HRH and EMMS management functions over time. This happened in response over time during that period with the progressive building of ***organizational structure and capacity*** at county level. From these findings, it is evident that decision space of decentralized units can be compromised by lack of capacity to undertake the decentralized functions.

**Table 2:** Shifting county level decision space over HRH and EMMS management functions corresponding to improvement in organizational structure and capacity of overtime

Function	County level decision space prior to July 2013	County level decision Space July – Dec 2013	County level decision space after December 2013
HRH Management			
Employment of staff	+	++++	++++
Deployment/distribution of staff	+	++++	++++
Payment of salaries	+	+	++++
EMMS Management			
Commodity quantification	+	++	++++
Commodity procurement	+	++	++++
Commodity allocation/distribution to health facilities	+	++	++++

In a study of health sector decentralization in Pakistan, Bossert and Mitchell (2011) reported that the de facto decision space over decentralized health sector management functions was always different from the *de jure* decision space; and that the difference was often due to the capacity of the individuals and institutions tasked to undertake the decentralized functions. Our findings agree with those of Bossert and Mitchell. They highlight the importance of ensuring that appropriate peripheral level capacity to undertake decentralized functions, is in place in decentralized units if the benefits of health sector decentralization are to be realized (Tsofa B, et al.: How does decentralisation affect health sector planning and financial management? A case study of early effects of devolution in Kilifi County, Kenya, submited) [33]. In addition, our findings also highlight the need to be ready to develop interim measures when this capacity is not yet available, and for central government and development partners to support this. The findings also highlight the need for clarity of roles of actors of different HRH and EMMS management functions across the different levels.

In our analysis, we note that the increase over time of county level decision space over HRH and EMMS management functions led to several positive effects. For HRH, the county gained the ability to determine the actual number of staff based on its budget and decide where to deploy them within the county. For EMMS, decentralized procurement led to a reported better fill-rate in health facilities, and the county was able to ensure all facilities were supplied with EMMS irrespective of registration status. This in turn allowed previously non-functioning facilities to operate, and thus previously underserved areas to have access to a facility. It is therefore likely that increased decision space at county level enhanced local level equity in the allocation of health resources, and health service provision at county level, and ensured services reached previously underserved populations.

However, the increase in county level decision space also led to perverse negative effects. With regards to HRH management, for example, the transfer to counties of decisions over the number and type of health workforce led to complications over transfers to other counties. In addition, the increase in county level decision space led to political interference over recruitment and deployment of staff, as local politicians began to demand that only health workers from within their county and tribe should be employed within the county. Political interference over HRH management within decentralized settings has also been reported in the Philippines by Grundy et al. (2003) [11]. Though not reported in our study, decentralized HRH recruitment has also been associated with inability to attract highly skilled health workers in rural remote areas in rural districts in Uganda [12]. Though our study was conducted in the early days of devolution implementation, this challenge may be less likely to occur in Kenya as the counties have more decision space for HRH management than their Ugandan counterparts, including the power to create special incentives to attract and retain staff.

For EMMS, as reported in Ghana and Guatemala [10], it could be argued that decentralized procurement led to the loss of economies of scale associated with a centralized procurement system. The increased decision space over EMMS management also fueled county level prioritization of the procurement of highly visible commodities such as ambulances at the expense of much needed drugs for Primary Health Care services (PHC). These observations are consistent with those made in Uganda and South Africa, where decentralized units prioritized allocations to curative services at the expense of PHC, as the former were more visible to the community [36–38]. This might be expected given the political nature of decentralization, leading local level political decision makers to prioritize issues that are more politically visible, and which will resonate with the electorate in order to maintain political support [39].

In relation to ***accountability structures and practices*** our study also found that these influenced decision space among health sector actors. For HRH, for example, we found that senior managers of the county government, including the County Department of Health, had to undergo public vetting at the County Assembly for their suitability for office, before being appointed. Similarly, we found that that the health facility managers had to involve their respective FMCs in the EMMS quantification process, and later inform them once the supplies are received at the facility. In both these cases, we find that the devolved government system was deliberately designed with increased public participation and accountability mechanisms with an intention to guard against potential decision space excesses by management decision makers. In addition to being an accountability mechanisms, enhanced public participation, especially in health resources allocation also does enhance community responsiveness of health service prioritization and thus promoting equity [22, 40].

In general, a recurring theme in the early days of Kenyan devolution affecting the design of the devolved government systems, the transfer process of county level functions generally, and the early effects on HRH and EMMS management process, was the politicization of the process. This observation underscores the arguments that by the mere fact that it involves the shifting of power and control from the center to the periphery, decentralization is a highly political process in its own right, and any attempt to analyse health sector effects of decentralization policies should always include an analysis of the political context [4, 7].

### Study strengths and limitations

The primary focus on only one county out of the 47 in the country could be considered a limitation of this study. However, the decision to use one county was deliberate, as it allowed for a deeper exploration of the issues under focus, by involving extended engagement with a broad range of stakeholders. Kilifi County is also part of the health systems governance learning sites for the Resilient and Responsive Health Systems (RESYST) consortium [21, 27]. This allows for longer-term tracking of the decentralization effects in this sites beyond the time of this study. The learning site setting also provided an opportunity for regular feedback to the county managers and national MoH thus increasing the potential of this study to inform the progressive implementation of devolution in the county and country.

## Conclusions

Decentralization has been an important element of the health system governance reform agenda for many years owing to its perceived importance in creating opportunities for strengthening local level management efficiency over ever-scarce health sector resources. For these reason, many health systems decentralization initiatives have included the goal of increasing local level decision space over management or resources.

The implementation of a devolved government system in Kenya has significantly increased county level decision space over HRH and EMMS management. This increased decision space created great potential in allowing for targeted recruitment and deployment of health workers, and procurement and distribution of EMMS based on local level priority needs. However, in practice this potential was undermined by organizational structure and capacity limitations, particularly in the early stages of implementation. Political interference also played a key role over HRH and EMMS management at county level, and was exacerbated by the combination of increased decision-space at sub-national levels at a time when structures and systems were not in place, and capacity was inadequate. That political interference was evident is hardly surprising, given that devolution itself is highly political, involving the transfer of power over management of public resources from national to sub-national levels of government.

### Recomendations

With these findings, we recommend the need for specific interventions to strengthen county level capacity over specific HRH and EMMS management functions so as to harness the potential positive effects of the increased decision space at county level brought about by the devolved government system in Kenya. These interventions should include creating clarity over HRH management roles between the CDoH and the CPSB, and improving the county payroll management system to stabilize payment of salaries for health care workers. On EMMS, there is need for strengthening the capacity of both health facility managers and CDoH managers in undertaking specifications and quantification of EMMS in order to streamline and speed up the ordering and procurement processes to avoid long periods of stock outs. At a national level, there is need for all stakeholders to come together to deliberate and build consensus on how certain HRH management roles including in-service training and inter-county transfers should be conducted across the country. For LMICs with similar settings to Kenya, we recommend that individual and institutional capacity considerations should always made when allocating functions between the center and the periphery during the design and implementation of health sector decentralization policies.
